# Depth-aware unpaired image-to-image translation for autonomous driving test scenario generation using a dual-branch GAN

**DOI:** 10.3389/fnbot.2025.1603964

**Published:** 2025-05-30

**Authors:** Donghao Shi, Chenxin Zhao, Cunbin Zhao, Zhou Fang, Chonghao Yu, Jian Li, Minjie Feng

**Affiliations:** ^1^Zhejiang Key Laboratory of Digital Precision Measurement Technology Research, Hangzhou, China; ^2^Advanced Manufacturing Metrology Research Center, Zhejiang Institute of Quality Sciences, Hangzhou, China; ^3^Key Laboratory of Acoustics and Vibration Applied Measuring Technology, State Administration for Market Regulation, Hangzhou, China

**Keywords:** autonomous driving, unpaired image-to-image translation, depth map, self-attention mechanism, generative adversarial network

## Abstract

Reliable visual perception is essential for autonomous driving test scenario generation, yet adverse weather and lighting variations pose significant challenges to simulation robustness and generalization. Traditional unpaired image-to-image translation methods primarily rely on RGB-based transformations, often resulting in geometric distortions and loss of structural consistency, which can negatively impact the realism and accuracy of generated test scenarios. To address these limitations, we propose a Depth-Aware Dual-Branch Generative Adversarial Network (DAB-GAN) that explicitly incorporates depth information to preserve spatial structures during scenario generation. The dual-branch generator processes both RGB and depth inputs, ensuring geometric fidelity, while a self-attention mechanism enhances spatial dependencies and local detail refinement. This enables the creation of realistic and structure-preserving test environments that are crucial for evaluating autonomous driving perception systems, especially under adverse weather conditions. Experimental results demonstrate that DAB-GAN outperforms existing unpaired image-to-image translation methods, achieving superior visual fidelity and maintaining depth-aware structural integrity. This approach provides a robust framework for generating diverse and challenging test scenarios, enhancing the development and validation of autonomous driving systems under various real-world conditions.

## 1 Introduction

Realistic and diverse test scenario generation is essential for evaluating autonomous driving systems, as perception models must effectively operate in complex and dynamic environments (Cai J. et al., 2024; Li X. et al., 2023; Zhang J. W. et al., 2024). This challenge becomes even more pronounced in adverse weather conditions, such as fog, rain, and low-light scenarios, which significantly impact object recognition, depth estimation, and overall scene understanding. These factors make it particularly difficult to assess the robustness and generalization ability of perception models, highlighting the need for realistic and varied test scenarios to ensure comprehensive evaluation.

Despite the importance of real-world testing, collecting diverse adverse weather data remains costly, time-consuming, and logistically challenging. Moreover, real-world datasets often suffer from imbalance and limited coverage of extreme conditions, restricting their utility in comprehensive validation and robustness assessment (Agarwal et al., 2025; Lan et al., 2024; Li Y. et al., 2023). As a result, simulation-based testing has become a critical tool for autonomous driving development, allowing for the controlled generation of challenging environmental conditions to enhance model reliability and adaptability (Biagiola and Tonella, 2024; Huang et al., 2025; Sadid and Antoniou, 2024). A key requirement for effective simulation is the ability to generate photorealistic and geometrically consistent test scenarios that accurately reflect real-world conditions.

Unpaired image-to-image translation has emerged as a powerful approach for scenario generation and domain adaptation, enabling the transformation of clear-weather images into adverse weather conditions without requiring paired training data (Ding et al., 2024; Wei et al., 2024). This technique is particularly valuable for augmenting training datasets and improving model robustness (Ye M. et al., 2024; Ye R. et al., 2024). However, traditional unpaired image-to-image translation methods primarily rely on RGB-based transformations, often resulting in geometric distortions and loss of structural consistency due to their pixel-wise nature, limiting their effectiveness for realistic autonomous driving test scenario generation.

Geometric consistency constitutes a fundamental principle for enhancing visual fidelity in autonomous driving simulation environments. In the domain of scene perception, the integration of depth information, edge-aware modules, and attention mechanisms has been widely recognized as an effective means of improving the performance of generative models (Tong et al., 2025a,b; Tong et al., 2024). Tong et al. (2025a) introduced an ORB-assisted sparse optical flow constraint as auxiliary guidance, which enhances the reliability of depth estimation in uncertain regions and significantly improves SLAM performance. Chen M. et al. (2024) proposed a novel framework that leverages depth distribution priors to regulate cross-domain mixing strategies and employs a multi-task Transformer-based fusion mechanism to enhance structural awareness, effectively mitigating scene layout inconsistencies in unsupervised domain adaptation. Collectively, these approaches underscore the importance of structural priors in geometry-aware modeling and provide valuable insights into addressing the limitations of CycleGAN in realistic scene generation for autonomous driving applications.

Inspired by the aforementioned studies, we explore the incorporation of depth information and attention mechanisms into the CycleGAN framework, we propose a Depth-Aware Dual-Branch Generative Adversarial Network (DAB-GAN) that explicitly incorporates depth information into the scenario generation process. By leveraging a dual-branch generator that simultaneously processes RGB and depth inputs, our approach enhances geometric fidelity and structural consistency in translated images. Additionally, a self-attention mechanism is integrated to refine spatial dependencies and local details, allowing for more realistic and structure-preserving transformations across diverse environmental conditions.

The contributions of this work are threefold:

1. Depth-Aware Dual-Branch Architecture: We introduce a novel generative model that processes RGB and depth information in parallel, ensuring geometric consistency during image translation.
2. Self-Attention Mechanism: By incorporating self-attention, our model enhances spatial coherence and improves the realism of translated images, particularly in adverse weather scenarios.
3. Superior Performance in Autonomous Driving Scenario Generation: Experimental results demonstrate that DAB-GAN surpasses existing unpaired image-to-image translation methods, achieving higher visual fidelity and preserving depth-aware structural integrity across diverse driving environments.

The structure of this paper is as follows: the second section provides a comprehensive review of related research on Depth-Aware Autonomous Driving Test Scenario Generation. The third section presents a detailed description of the proposed method, highlighting its integration with the dual-branch architecture and self-attention mechanism. The fourth part reports the experimental results and performance evaluations, while the fifth part concludes the paper with key findings and potential directions for future research.

## 2 Related works

### 2.1 Generative adversarial networks for image translation

GANs (Goodfellow et al., 2014) have significantly advanced image synthesis and translation, broadly categorized into unconditional GANs and conditional GANs.

Unconditional GANs learn to generate images of a specific category solely from input images, without requiring explicit conditional guidance. Radford et al. (2016) introduced convolutional architectures that improved training stability and image quality, while StyleGAN (Karras et al., 2020, 2021a,b) further refined image synthesis through a style-based architecture, enabling fine-grained control over image attributes and facilitating high-resolution image generation.

Conditional GANs, in contrast, leverage auxiliary information to guide the image generation process and can be further classified into paired image-to-image translation and unpaired image-to-image translation (Chen L. et al., 2024; Shubham et al., 2024). While paired image-to-image translation has demonstrated significant advancements across various applications, its reliance on paired training data poses a major challenge due to the high cost and limited availability of such datasets in real-world scenarios (Liu M. et al., 2017; Shrivastava et al., 2017; Zhu et al., 2017). To address this limitation, unpaired image-to-image translation methods have been developed, enabling models to learn and transfer styles between different domains without the need for explicitly paired samples (Cai X. et al., 2024; Lin et al., 2025; Zhu et al., 2017). Liu M. et al. (2017) introduced CoGAN, which establishes a coupling relationship between generated images and style images, allowing them to interact and influence each other during training. CycleGAN (Zhu et al., 2017), on the other hand, proposed a cycle-consistency constraint, ensuring that an image translated from domain A to B can be accurately reconstructed back to A. This self-supervised learning framework effectively preserves structural and semantic consistency without the requirement for paired data, making CycleGAN a widely adopted baseline for unpaired image translation tasks (Liu et al., 2024; Yang L. et al., 2024; Ye H. et al., 2024).

Building on the success of CycleGAN, this work integrates depth information and self-attention mechanisms to further enhance structural preservation, semantic coherence, and visual fidelity in unpaired image-to-image translation, with a specific focus on autonomous driving test scenario generation. By leveraging these enhancements, our approach aims to improve the realism and consistency of generated test environments, facilitating more robust evaluations of autonomous driving perception systems under diverse conditions.

### 2.2 Depth map utilization in image synthesis

Depth information is pivotal in scene understanding, offering geometric constraints that enhance spatial consistency during image translation (Ming et al., 2021; Piccinelli et al., 2024). While depth-aware processing has been extensively explored in applications such as 3D reconstruction, depth estimation, and image super-resolution, its integration into unpaired image-to-image translation remains underexplored. Prior research has primarily focused on monocular depth estimation and stereo image synthesis, utilizing depth maps as auxiliary inputs to bolster structural consistency (Hong et al., 2024; Hong et al., 2022). However, within the realm of GAN-based translation, the incorporation of depth maps into the generative process is seldom addressed.

Recent studies have begun to explore depth-guided generative models for style transfer and domain adaptation. Liu X. et al. (2017) employed depth maps to preserve spatial layout during style transfer, treating depth as a supplementary constraint, and achieved promising results. Building upon this idea, Ioannou and Maddock (2022) utilized an advanced depth prediction network to incorporate depth preservation as an additional loss alongside content and style losses. Experiments demonstrated that their proposed method outperformed other models in comparative evaluations. Similarly, Hong et al. (2022) proposed DaGAN and its improved version, DaGAN++ (Hong et al., 2024), which integrate depth information to guide facial keypoint detection and cross-modal attention learning in talking head video generation. These works highlight the potential of incorporating depth into generative tasks. However, such approaches often treat depth information as a secondary feature rather than as a core component of the image transformation process.

In contrast, our proposed dual-branch GAN architecture explicitly processes depth maps alongside RGB images, enabling the model to jointly learn appearance-based style modifications and depth-preserving geometric structures.

### 2.3 Attention mechanisms in GANs

In traditional Generative Adversarial Networks (GANs), convolutional layers are inherently limited by their local receptive fields, making it challenging for the generator to simultaneously capture global structures and preserve fine-grained details. Attention mechanisms, on the other hand, have demonstrated remarkable effectiveness in enhancing image generation quality by capturing long-range dependencies and refining intricate details (Xue et al., 2024; Ye and Wang, 2024). Due to their ability to selectively focus on important regions of an image while maintaining contextual coherence, attention mechanisms have been widely adopted in GAN-based image synthesis, particularly for refining style transfer and enhancing content preservation.

Bakht et al. (2024) incorporated Multi-Level Attention into a GAN framework to improve underwater image restoration, effectively preserving textures, edges, and object structures. Experimental evaluations demonstrated the superior performance of their approach, further validating its efficacy in underwater scene analysis. Yildiz et al. (2024) integrated self-attention into a GAN-based single-image generation framework, with extensive evaluations across multiple datasets confirming significant improvements in image quality, training stability, and texture preservation, outperforming existing models. These studies highlight the transformative impact of attention mechanisms in image generation, particularly in scenarios requiring fine-detail preservation and structural coherence.

Given the demonstrated effectiveness of attention mechanisms in GAN-based image generation, we extend CycleGAN by incorporating self-attention into its generator network. This integration allows the model to dynamically prioritize spatially significant regions while maintaining global style consistency, thereby enhancing structural coherence, semantic integrity, and overall visual realism in unpaired image-to-image translation.

### 2.4 Image translation for autonomous driving test scenario generation

The generation of realistic and diverse test scenarios is essential for the development and validation of autonomous driving systems. Traditional methods, such as real-world driving and closed-course simulations, often fail to capture the full range of traffic situations and rare edge cases that autonomous vehicles may encounter (Li et al., 2022; Zhang J. W. et al., 2024). To address these limitations, image translation techniques have emerged as a promising solution for generating diverse and comprehensive test scenarios.

Image translation refers to methods that transform images from one domain to another, such as converting a clear road scene into foggy or nighttime conditions. This capability is particularly valuable for autonomous driving, as it enables the simulation of various environmental scenarios, such as different weather conditions and lighting, which can significantly enhance the testing of vehicle perception systems (Lambertenghi and Stocco, 2024). Lakmal et al. (2024) tackled the challenge of night-to-day image translation in autonomous driving, where acquiring paired night-day images in real-world settings is inherently difficult. To address this, they first employed CycleGAN to synthesize a low-quality paired dataset, which served as the training data for their proposed NtD-GAN model. This model demonstrated improved translation quality over existing approaches. However, its generalization capability remains limited, particularly under diverse and complex driving conditions such as rain, fog, and low-visibility scenarios.

Meanwhile, many researchers have explored the use of unpaired image translation techniques to enhance model generalization. By employing unpaired image translation, autonomous vehicles can be trained and tested in a broad range of simulated environments without the need for paired datasets, thus reducing the complexity and cost associated with data collection (Lan et al., 2024; Zhao et al., 2024). Lee et al. (2022) proposed a method that uses image translation to simulate weather effects, such as rain and fog, by transforming LiDAR data between different weather scenarios. This approach facilitates the creation of synthetic data, which augments training datasets and enhances a vehicle’s ability to handle challenging environmental conditions, thereby improving the robustness of autonomous driving systems. Fernando et al. (2024) addressed the challenge of limited visibility in low-light conditions for autonomous driving systems by proposing a CycleGAN-based framework that integrates RGB and infrared (IR) imagery to perform night-to-day image translation. Operating within an unsupervised learning paradigm, the model effectively circumvents the need for labor-intensive paired datasets. Similarly, El Madawi et al. (2019) investigated the fusion of RGB images and LiDAR data for 3D semantic segmentation, leveraging the complementary nature of camera-derived color information and LiDAR-based depth measurements to enhance object recognition accuracy. The aforementioned approaches utilize unpaired image translation techniques for autonomous driving scene generation, introducing IR or LiDAR modalities into a CycleGAN baseline to impose additional structural constraints on the generative adversarial network, thereby improving the realism and fidelity of the synthesized scenes. However, the reliance on auxiliary modalities such as IR or LiDAR inevitably increases data acquisition complexity and cost, which can impose a substantial burden in the context of large-scale scenario generation for autonomous driving system testing.

Therefore, this study adopts an unpaired image translation network as the baseline to enhance the model’s generalization performance. At the same time, it investigates whether high-quality autonomous driving scenario generation can be achieved without incurring additional data acquisition costs. To this end, we propose a dual-branch architecture based on CycleGAN, which integrates monocular depth estimation into the original RGB images, thereby improving both the visual quality and structural integrity of the translated scenes.

## 3 Main work

In this section, we first describe the two-branch network structure with the original image and its depth map as input, and explain the depth map generation method. We use a dual-branch generator and discriminator, incorporating adversarial loss and cycle consistency loss to constrain the training, thereby achieving style transfer between different datasets. Additionally, we provide details of the generator and discriminator, including the module composition and input–output channel parameters of both networks. Finally, we explain the principle and composition of the added self-attention mechanism.

### 3.1 Dual-branch network architecture with depth information

In this study, we aim to learn two domain translation functions, referred to as GeneratorA and GeneratorB, which are responsible for mapping an input image realA, along with its corresponding depth map depthA, to a synthetic image fakeA. The objective is to ensure that fakeA not only preserves the spatial and structural consistency inherent in realA, but also effectively adopts the stylistic characteristics of the target domain realB. The overall framework of the network is shown in Supplementary Figure 1.

As an initial step, we employ the Depth Anything model to estimate depth information from real-world input images, serving as a means to provide geometric guidance for subsequent translation tasks. Depth Anything (Yang S. et al., 2024) trains a robust monocular depth estimation model using large-scale unlabeled datasets, and it has been shown to be state-of-the-art among a large number of monocular depth estimation models.

After obtaining the depth map, we input both the real image realA and its corresponding depth map depthA into GeneratorA to produce the translated image fakeA. To improvice the quality of fakeA, we employ DiscriminatorA, which takes both fakeA and realA as input and computes the adversarial loss $L_{\text{adversarial}1}$.

In addition, we use the pre-trained GeneratorB to reconstruct the input by feeding it fakeA and depthA, yielding recA. The cycle consistency loss is then computed as the expected pixel-wise difference between recA and realA. Similarly, we obtain the identity loss $L_{\mathrm{idt}1}$ by passing realA and depthA into GeneratorB to generate idtA, and computing the expectation of pixel-wise differences between idtA and realA.

All loss terms are backpropagated to optimize GeneratorA. The design and training procedure for GeneratorB follow the same principle in the reverse direction.

### 3.2 Generator and discriminator details

Generator is a dual-branch network composed of two identical networks, the framework of G is shown in Supplementary Figure 2A, and the detailed architectures of the branches can be found in Supplementary Table 1. Each branch consists of feature extraction layers, ResNet block layers, self-attention layers, and upsampling layers. The feature extraction layers contain three convolutional layers, which are used to extract features from the input and reduce the image size. The ResNet block layers consist of 9 ResNet blocks, which are used to further extract information from the input features. The self-attention layer introduces a self-attention module to enhance the model’s focus on key information in the input image. Finally, we use upsample layers to increase the image size through the ConvTranspose module and a convolutional module. Additionally, using the residual connection, we add the outputs of the two networks and divide by 2 to obtain the output $Y_{\text{fake}}$.

As for the discriminator, its structure is identical to that of the PGGAN discriminator (Karras et al., 2017). This choice is motivated by PatchGAN’s effectiveness in focusing on high-frequency structure, as it operates on local image patches (typically 70 × 70 receptive fields), making it well-suited for capturing texture and style-level details rather than global image semantics. The framework of discriminator is shown in Supplementary Figure 2B, and the detailed architectures of the branches can be found in Supplementary Table 2.

Structurally, the discriminator is implemented as a convolutional neural network composed of multiple layers of strided convolutions, each followed by a LeakyReLU activation function. The number of feature maps increases progressively with network depth, typically doubling at each subsequent layer until reaching a maximum of 512 channels. Instead of producing a single scalar output for the entire input image, the network generates a one-channel feature map, where each spatial location represents the likelihood that the corresponding image patch is real or fake. This patch-based prediction mechanism encourages the generator to produce visually convincing fine-grained details across the entire image, thereby improving the overall realism of the synthesized outputs.

### 3.3 Implementation details of the depth map module

In the field of unpaired image-to-image translation, to the best of our knowledge, there is currently no method that explicitly utilizes depth map information as input to constrain the translation process. The absence of depth constraints often results in the loss of structural information in the generated images, leading to phenomena such as mode collapse and distortion. Incorporating depth map information can effectively enhance the quality of model training and improve the structural consistency of generated outputs. Therefore, we propose to integrate depth maps as part of the input to our framework.

Our depth estimation method is based on the monocular depth estimation approach proposed in Depth Anything, as illustrated in Supplementary Figure 3. Specifically, we utilize the pretrained model released by Depth Anything to generate depth maps for the input images without making any modifications. The depth map module is designed to leverage both labeled and unlabeled images for training. The labeled dataset and unlabeled dataset are denoted as $D_{l}={\left\{(x_{i},d_{i})\right\}}_{i=1}^{M}$ and $D_{u}={\left\{u_{i}\right\}}_{i=1}^{N}$, respectively. In the first stage, a teacher model T is trained on the labeled dataset using an affine-invariant depth loss to account for variations in scale and shift. The affine-invariant loss between the predicted depth $\hat{d_{i}}$ and the ground truth depth $d_{i}$ is formulated as:

$$L_{\text{affine}}=\frac{1}{\mathrm{HW}}\sum_{i=1}^{\mathrm{HW}}|\hat{d_{i}^{*}}-\hat{d_{i}}|$$

where the predicted and ground truth depths are normalized by subtracting the median and dividing by the mean absolute deviation to eliminate global scale and shift differences.

The trained teacher model is then used to generate pseudo-depth labels for the unlabeled dataset. In the second stage, a student model S is trained by combining labeled samples and pseudo-labeled unlabeled samples, enhancing generalization across diverse scenes.

It is important to note that the teacher and student models share an identical network architecture. The encoder is initialized with a pretrained DINOv2 (Oquab et al., 2023) ViT-L model that extracts multi-scale feature representations, and the decoder consists of a DPTHead module that reconstructs dense depth maps from these intermediate features. The overall framework of the model is shown in Supplementary Figure 4. To further improve the quality of the generated depth maps, two types of strong perturbations are introduced during the student training phase: color-based distortions such as color jittering and Gaussian blur, and spatial distortions implemented via CutMix (Yun et al., 2019), which is formulated as:


$$u_{\mathrm{ab}}=u_{a}⊙M+u_{b}⊙(1-M)$$

Where M is a binary mask and ⊙ denotes element-wise multiplication.

Additionally, to enhance semantic understanding and improve feature robustness, a feature alignment loss is applied between the features extracted by the depth encoder and the features extracted by a frozen DINOv2 model. This loss encourages the student model to inherit high-level semantic priors and is defined as:


$$L_{\text{feat}}=1-\frac{1}{\mathrm{HW}}\sum_{i=1}^{\mathrm{HW}}\cos(f_{i},f_{i}^{\prime })$$

Where $f_{i}$ and $f_{i}^{\prime }$ denote the feature vectors from the student model and the frozen DINOv2 model at pixel i, respectively.

Finally, semantic segmentation labels are assigned to the unlabeled images to provide additional supervision. These semantic annotations are generated by combining predictions from RAM (Zhang Y. et al., 2024), GroundingDINO (Liu et al., 2025), and HQ-SAM (Ke et al., 2023), further enhancing the semantic representation learned by the model.

### 3.4 Self-attention module

Attention mechanisms have been widely applied to various computer vision tasks. Compared to traditional convolutional networks, attention mechanisms are capable of capturing global information from input data, such as extracting contextual relationships in natural language processing tasks, while also focusing on important regions within a broader context (Devlin et al., 2019).

In this paper, we incorporate a self-attention module, with its specific structure illustrated in Supplementary Figure 5. As shown in the figure, the self-attention module consists of two stages, where feature extraction and information fusion are applied to the output of the ResNet block.

In Stage I, the input feature map $x\in {\mathbb{R}}^{C\times H\times W}$ is transformed through three 1×1 convolutional layers to generate the query matrix Q, key matrix K, and value matrix V. The transformation is formulated as:


$$Q=\mathrm{Con}v_{1\times 1}^{\left(q\right)}(x)$$

$$K=\mathrm{Con}v_{1\times 1}^{\left(k\right)}(x)$$

$$V=\mathrm{Con}v_{1\times 1}^{\left(v\right)}(x)$$

In Stage II, similarity matching is performed between the query and key matrices to compute attention weights, which are then fused with the value matrix to obtain the final output of the self-attention module. The matrix operation for Stage II is expressed as follows:


$$\text{Self}\_\text{attention}(Q,K,V)=\text{Softmax}(\frac{QK^{T}}{\sqrt{d_{K}}})V$$

Among them, Q is the query matrix, K is the key matrix, V is the value matrix, $d_{K}$ is the dimension of matrix K, the three matrices have variables are divided into the head, the use of linear transformation conversion.

## 4 Dataset and evaluation

### 4.1 Experimental data

For this study, we constructed a custom dataset comprising road scene imagery captured using dashcams, as shown in Supplementary Figure 6. The top two rows depict examples from the source domain dataset, which includes elements commonly found in autonomous driving scenarios such as pedestrians, bicycles, delivery vehicles, sedans, and buses. This dataset was collected using a dashcam and captures a variety of road conditions including urban streets and highways. It consists of a total of 6,480 images and forms the original dataset, denoted as X.

The middle two rows show sample images from the target domain dataset, which also include various scenes and elements from everyday life. This dataset is derived from the open-source Foggy Cityscapes dataset (Cai et al., 2019) and contains a total of 3,457 foggy images. It is used as the style dataset, denoted as Y.

In addition, we further utilized the open-source KITTI dataset (Geiger et al., 2012) as another source domain dataset. Corresponding foggy versions of the images were generated to evaluate the generalization ability of our method. The bottom two rows in Supplementary Figure 6 display sample images from the KITTI dataset, which contains a total of 7,071 images.

### 4.2 Evaluation metrics

#### 4.2.1 Methods for comparison

We compare our method with five existing approaches and conduct a series of experiments, including ablation studies, hyperparameter comparisons, visual comparisons of the generated images, and visual comparisons of the corresponding depth maps. The methods in our comparative evaluation are the follows:

- DCGAN (Radford et al., 2016) stands for Deep Convolutional Generative Adversarial Network, which utilizes convolutional layers to improve the stability and quality of image generation in GANs.
- CoGAN (Liu and Tuzel, 2016) consists of two GANs that share weights to learn a joint distribution of multi-domain images, enabling generation of corresponding images in different domains.
- Stylegan2 (Karras et al., 2020) is a state-of-the-art generative adversarial network for producing highly realistic and high-resolution images through advanced style-based architecture.
- Pix2Pix (Isola et al., 2017) is a conditional generative adversarial network designed for image-to-image translation tasks, capable of transforming images from one domain to another using paired training data.
- Cyclegan (Zhu et al., 2017) is a generative adversarial network designed for unpaired image-to-image translation, allowing conversion between two image domains without needing paired examples.

#### 4.2.2 Metrics

We choose PSNR (Peak Signal-to-Noise Ratio), SSIM (Structural Similarity) and FID (Fréchet Inception Distance) as quantitative indicators.

PSNR is an index used to measure the effect of image compression or denoising, which evaluates the image quality by calculating the mean square error (MSE) between the original image and the compressed or processed image. It is defined as:


$$\mathrm{PS}NR=10\cdot \log_{10}(\frac{\mathrm{MA}X_{I}^{2}}{\mathrm{MSE}})$$

Where ${\mathrm{MAX}}_{I}$ is the maximum possible pixel value of the image (typically 255), and MSE is the mean squared error between the generated image and the reference image. The larger the PSNR value, the better.

SSIM is used to evaluate the structural similarity of two images. It takes into account the brightness, contrast and structure information of the image, which is closer to the perception of image quality by the human eye. The SSIM is computed as:


$$\text{SSIM}(x,y)=\frac{\left(2\mu_{x}\mu_{y}+C_{1})(2\sigma_{\mathrm{xy}}+C_{2}\right)}{\left(\mu_{x}^{2}+\mu_{y}^{2}+C_{1})(\sigma_{x}^{2}+\sigma_{y}^{2}+C_{2}\right)}$$

Where $\mu_{x}$, $\mu_{y}$ are the average values, $\sigma_{x}^{2}$, $\sigma_{y}^{2}$ are the variances, and $\sigma_{\mathrm{xy}}$ is the covariance of images x and y. $C_{1}$ and $C_{2}$ are constants to stabilize the division. The larger the SSIM, the better.

FID is a metric used to evaluate the quality of the generated image and is often used to evaluate the performance of GANs. It measures quality by calculating the difference between the distribution of the generated image and the real image in the feature space. The formula is:


FID=‖μr−μg‖2+Tr(Σr+Σg−2(ΣrΣg)12)

Where ($\mu_{r},\sum_{r}$) and ($\mu_{g},\sum_{g}$) are the means and covariances of the real and generated image features, respectively. A smaller FID value indicates a higher similarity to real images and thus better performance.

### 4.3 Experimental verification

#### 4.3.1 Training strategy and loss function

During the training process, input images are initially resized to a resolution of 286 pixels and subsequently center-cropped to 256 pixels to conform to the input dimensional requirements of the network. To enhance data diversity and improve the model’s generalization capability, particularly with respect to unseen perspectives and spatial configurations, random horizontal flipping is applied by default.

More critically, CycleGAN adopts an asymmetric update strategy to balance the optimization dynamics between the generator and the discriminator. Given that the generator is tasked with learning complex high-dimensional mappings, its training is inherently more challenging, especially during the early phases of learning. In contrast, the discriminator tends to converge more rapidly, which may lead to overly confident predictions and result in vanishing gradients for the generator. To mitigate this imbalance and stabilize the adversarial training process, the update frequency is carefully controlled: the generator is updated three times for every single update of the discriminator.

To ensure both realism and geometric consistency in the unpaired image translation task, particularly under adverse conditions for autonomous driving scenarios, our model employs a composite loss function that integrates adversarial learning, cycle consistency, identity preservation, and depth-aware constraints. These loss components collectively enable robust style transfer while maintaining structural fidelity, which is critical for simulating realistic driving environments.

The total objective function of the proposed Dual-Branch Depth-Aware GAN is expressed as:


$$\begin{array}{l} L_{\text{total}}=L_{\mathrm{adv}}(G,D_{B},X_{A},X_{B})+L_{\mathrm{adv}}(F,D_{A},X_{B},X_{A})+\\ \text{lambda}L_{\mathrm{cyc}}(G,F)+\text{Identity}L_{\mathrm{idt}}(G,F) \end{array}$$

Where G:A→B and F:B→A, denote the forward and backward generators, respectively. $X_{A}$ denotes the source domain datasets and $X_{B}$ denotes the target domain datasets. $D_{A}$ and $D_{B}$ are the corresponding discriminators. lambda and Identity are hyperparameters controlling the strength of the cycle consistency and identity losses.

To encourage the generators to produce outputs that are indistinguishable from the real images of the target domain, we adopt the least-squares adversarial loss (LSGAN), which has been shown to stabilize training and generate higher-quality results. For the generator G and discriminator $D_{B}$, the loss is defined as:


$$\begin{array}{l} L_{\mathrm{adv}}(G,D_{B},X_{A},X_{B})=E_{x_{b}\sim p_{\text{data}}(x_{b})}[{\left(D_{B}(x_{b})-1\right)}^{2}]+\\ E_{x_{a}\sim p_{\text{data}}(x_{a})}[D_{B}{\left(G(x_{a})\right)}^{2}] \end{array}$$

To enforce that the learned mappings are bijective and to preserve semantic structure, we use a cycle consistency loss. This loss ensures that an image translated from one domain to another and then back should closely resemble the original input. It is given by:


$$\begin{array}{l} L_{\mathrm{cyc}}(G,F)=E_{x_{a}\sim p_{\text{data}}(x_{a})}[{||F(G(x_{a}))-x_{a}||}_{1}]+\\ E_{x_{b}\sim p_{\text{data}}(x_{b})}[{||G(F(x_{b}))-x_{b}||}_{1}] \end{array}$$

To regularize the mapping and maintain color and content consistency, particularly in scenarios where images are already in the target domain, we adopt an identity loss:


$$\begin{array}{l} L_{\mathrm{idt}}(G,F)=E_{x_{b}\sim p_{\text{data}}(x_{b})}[{||G(x_{b})-x_{b}||}_{1}]+\\ E_{x_{a}\sim p_{\text{data}}(x_{a})}[{||F(x_{a})-x_{a}||}_{1}] \end{array}$$

This encourages the generators to behave as identity mappings when fed images from the target domain, improving semantic fidelity in the translated outputs.

#### 4.3.2 Comparison against other methods

As shown in Supplementary Table 3, the performance of our proposed method and five comparison methods is evaluated using three quantitative metrics on the custom-designed dataset specifically curated for this study. The results indicate that our approach consistently outperforms the baselines across all evaluation indicators. In particular, the Structural Similarity Index Measure (SSIM) of the images generated by our method reaches 0.80, which is significantly higher than that of the other methods, demonstrating superior image quality and structural preservation.

In addition to the quantitative results, we provide qualitative comparisons of the generated images, as illustrated in Supplementary Figure 7. The leftmost column displays the original input images, the middle columns show the results produced by the baseline methods, and the rightmost column presents the images generated by our proposed method. From the figure, it can be observed that DCGAN and CoGAN struggle to generate coherent and realistic road scene images. While StyleGAN2 and Pix2Pix offer moderate improvements, they often produce mismatches and distortions due to a lack of strong constraints on image structure. CycleGAN shows further enhancement in visual quality, but still suffers from issues such as local artifacts, element distortion, and the unintended addition of scene elements.

In contrast, our method integrates depth map information and a self-attention mechanism into the generation process, which effectively mitigates the above issues. It better preserves semantic elements from the original images, accurately captures scene structure, and significantly reduces artifacts and distortions during translation. These results validate the effectiveness of our design in enhancing both the visual realism and structural consistency of translated images.

#### 4.3.3 Ablation experiment

To evaluate the effectiveness of our framework design, we conduct a comprehensive ablation study using the custom-designed dataset. Specifically, we remove individual components from the proposed method one at a time to assess their respective contributions. The ablated versions of our method are defined as follows:

- m/oself-att: The proposed method without the self-attention component.
- m/odepth: The proposed method without the depth map and dual-branch network structure.
- m/oboth: The proposed method without the depth map, dual-branch network structure and self-attention component.
- m: The proposed method.

The test results, evaluated using PSNR, SSIM, and FID metrics, are presented in Supplementary Figure 8. Several key observations can be drawn from these results:

1. In terms of PSNR and SSIM, our proposed method achieves the best experimental performance. For FID, while our method also shows significant improvement, it ranks second, slightly behind the model incorporating the self-attention mechanism, with a minor gap between the two approaches.
2. Regarding PSNR, the addition of both depth map and self-attention improves the generated image quality, with the depth map having a more pronounced effect. For SSIM, the improvement from both additions is similar. In contrast, the FID score benefits more from the inclusion of the self-attention mechanism.
3. As indicated by the results, incorporating the depth map is particularly effective in image denoising, enhancing the realism and stability of the generated images. Meanwhile, the self-attention mechanism is more effective in capturing the distribution characteristics and structural information of the images.

The experimental results demonstrate that the framework we designed outperforms others across all evaluation metrics. The attention mechanism, known for expanding the receptive field and improving the algorithm’s ability to extract crucial features, contributes significantly to the enhancement of the model’s performance. The dual-branch structure with added depth map information plays a crucial role in providing effective constraints to the generation network, allowing it to produce images consistent with the depth map, which improves the overall quality of the generated output.

To validate our hypothesis, we provide result diagrams and generated images comparing the proposed method with the dual-branch network method without depth map information, as shown in Supplementary Figure 9. From the figure, it is evident that our proposed method preserves the road elements well, while the method without depth maps fails to maintain background road features effectively. Moreover, the latter may introduce elements not present in the original road scene, such as the addition of a car logo in the original image X, or the appearance of a car in the middle of the road in image Y. Additionally, the vehicle shape in the original image Y becomes distorted.

From the experimental results, it is clear that the dual-branch network incorporating depth map information better maintains the features of road elements and effectively prevents issues such as distortion, false shadows, and other artifacts in the generated images.

#### 4.3.4 Hyperparameter comparison

We have compared different hyperparameters of the network, and the experimental results are shown in Supplementary Table 4. n_epochs represents the number of iterations in which the learning rate does not decay at the beginning of the training process, and n_epochs_decay represents the number of iterations in which the learning rate decays after the training epochs round. It can be seen from the table that applying learning rate decay significantly affects model training performance. In this problem, n_epochs = 50, n_epochs_decay = 50, the generation effect is the best. lambdaA and lambdaB represent the weights of the generator and discriminator during training, respectively, and the best effect is achieved when lambdaA = 100 and lambda = 100. Identity indicates the weights of Indentity_loss and reconstruction loss in the training process. As can be seen from the table, the best result is generated when Identity = 0.5.

#### 4.3.5 Presentation of results

Supplementary Figure 10 illustrates the results of road scene generation using the proposed model using the custom-designed dataset. The first row displays the original images; the second row shows the corresponding depth maps generated by the Depth Anything model; the third row presents samples from the style dataset Y, selected from the Foggy Cityscapes dataset; and the final row shows the foggy images generated by our method.

As observed from the figure, the images produced by our model not only effectively preserve the semantic elements and structural integrity of the original road scenes. Such as road markings, vehicles, and surrounding objects, but also successfully learn and replicate the foggy style characteristics of the target domain Y. This demonstrates the model’s strong ability in both content preservation and style transfer.

To ensure the objectivity of the evaluation, the same set of source images was used across all methods, and visual comparisons were supplemented with quantitative metrics as presented in previous sections. The consistency between the learned depth structure and the transferred style highlights the advantage of integrating depth information in the generative process.

In addition, we further employed the open-source KITTI dataset as the source domain dataset to generate foggy-style images using the pretrained weights obtained from our model trained on the custom dashcam dataset. The generation results are presented in Supplementary Figure 11. As shown in the figure, the proposed method successfully applies fog-style effects to the original scenes while preserving the underlying structural content. These results not only demonstrate the effectiveness of the style transfer but also validate the generalization capability of the model across different datasets.

## 5 Conclusion

Reliable visual perception is essential for autonomous driving, as accurate scene understanding is crucial for safe decision-making in dynamic and challenging environments. However, adverse weather conditions such as fog, rain, and low-light environments introduce significant challenges to the robustness and generalization of perception models. Traditional unpaired image-to-image translation methods, which primarily rely on RGB-based transformations, often lead to geometric distortions and loss of structural consistency. These issues are especially problematic in safety-critical applications such as autonomous driving. To address these challenges, we propose the Depth-Aware Dual-Branch Generative Adversarial Network (DAB-GAN), which integrates depth information directly into the generative process. This approach preserves spatial structures and enhances translation accuracy in adverse conditions.

Our DAB-GAN model utilizes a dual-branch generator that processes both RGB and depth inputs, ensuring geometric fidelity while leveraging a self-attention mechanism to enhance spatial dependencies and refine local details. This integration allows for the generation of more realistic and structurally coherent images that are vital for autonomous driving perception systems. The incorporation of depth-aware structural integrity significantly improves the generalization capabilities of vision-based models, particularly in dynamic environments and challenging weather conditions.

Through extensive experiments, we demonstrate that DAB-GAN outperforms existing unpaired image-to-image translation methods across multiple benchmarks, achieving superior visual fidelity and structural consistency. Our results validate the effectiveness of combining depth-awareness and self-attention mechanisms to improve the quality and realism of generated images, ensuring that the translated images remain both visually realistic and geometrically faithful.

This work highlights the potential of depth-aware generative models to address real-world challenges in autonomous driving systems. Future work will explore optimization techniques for real-time applications, as well as the fusion of multi-sensor data to further enhance translation accuracy and system robustness in real-world autonomous driving deployments.

## Data Availability

Publicly available datasets were analyzed in this study. This data can be found at: 18323135471@163.com to get the data.
