# Supplementary material for: Depth-aware unpaired image-to-image translation for autonomous driving test scenario generation using a dual-branch GAN
Source: Front Neurorobot. 2025 May 30;19:1603964. doi: 10.3389/fnbot.2025.1603964 (PMC12162506; doi:10.3389/fnbot.2025.1603964)
Supplement: Supplementary file 1 [file Data_Sheet_1.docx]

Supplementary Material

# Supplementary Figures and Tables

## Supplementary Figures


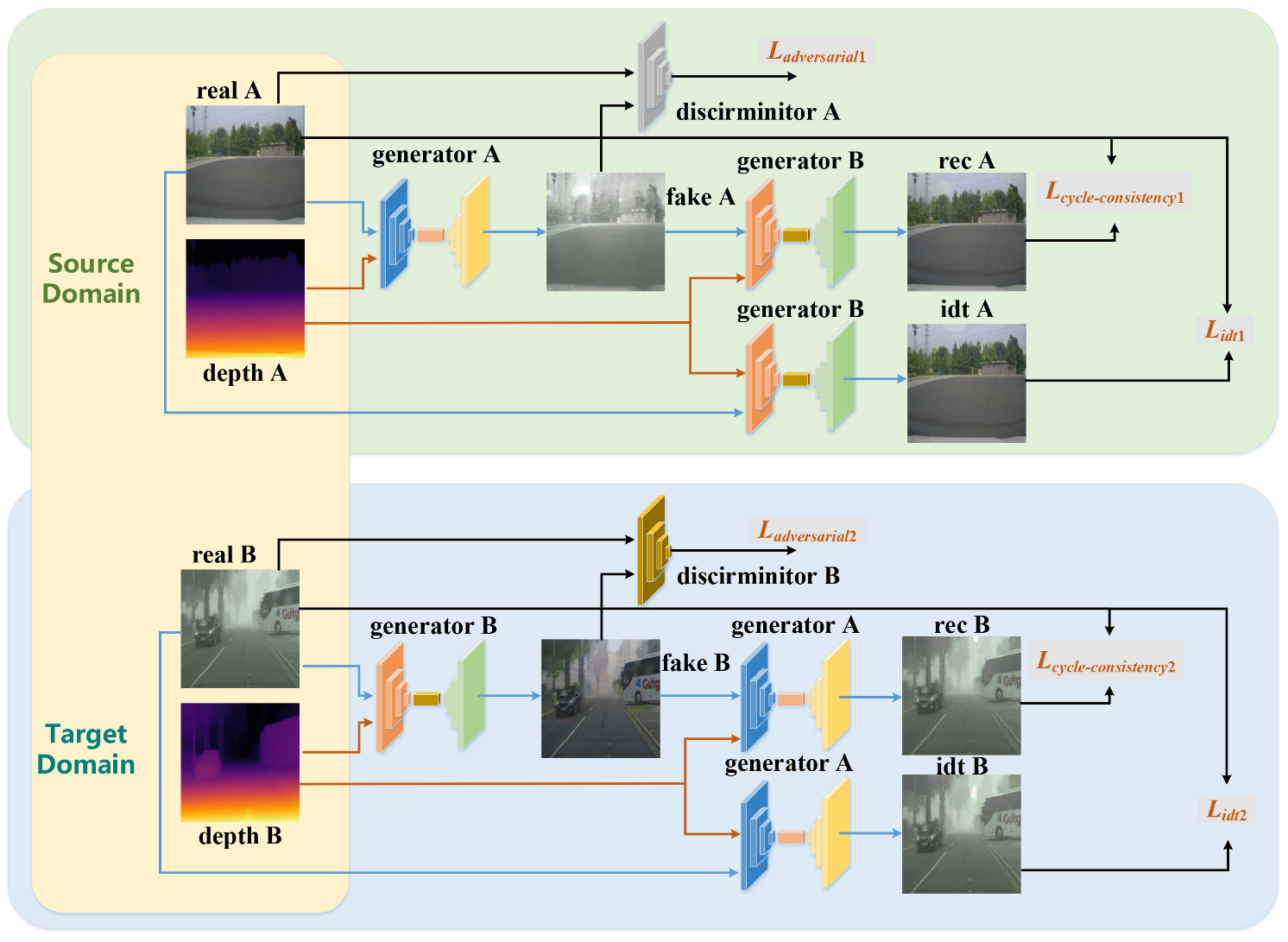


**Supplementary Figure 1. Structural detail diagram of our proposed method.**


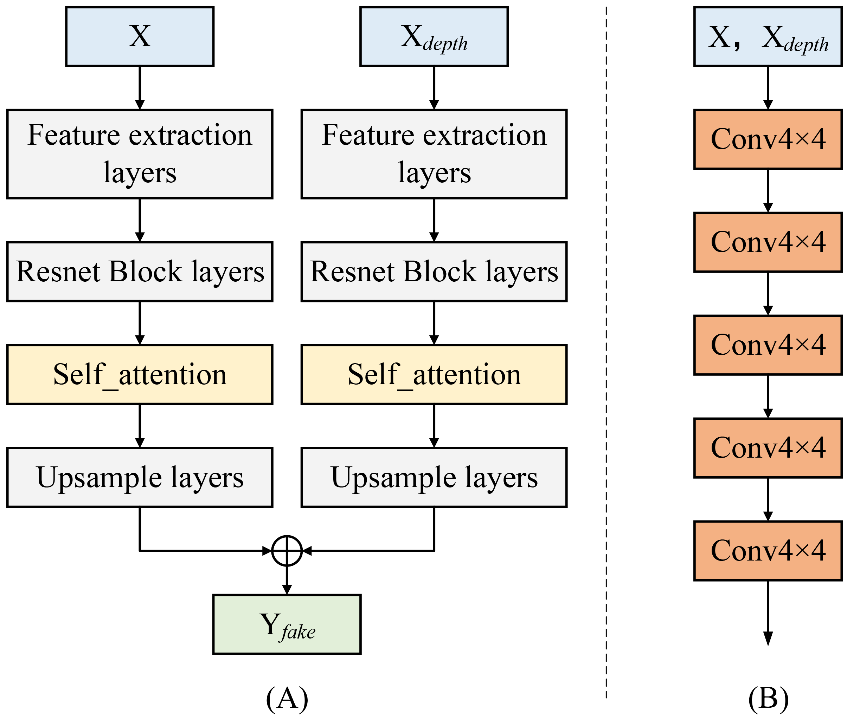


**Supplementary Figure 2. The structure of our proposed generator.**


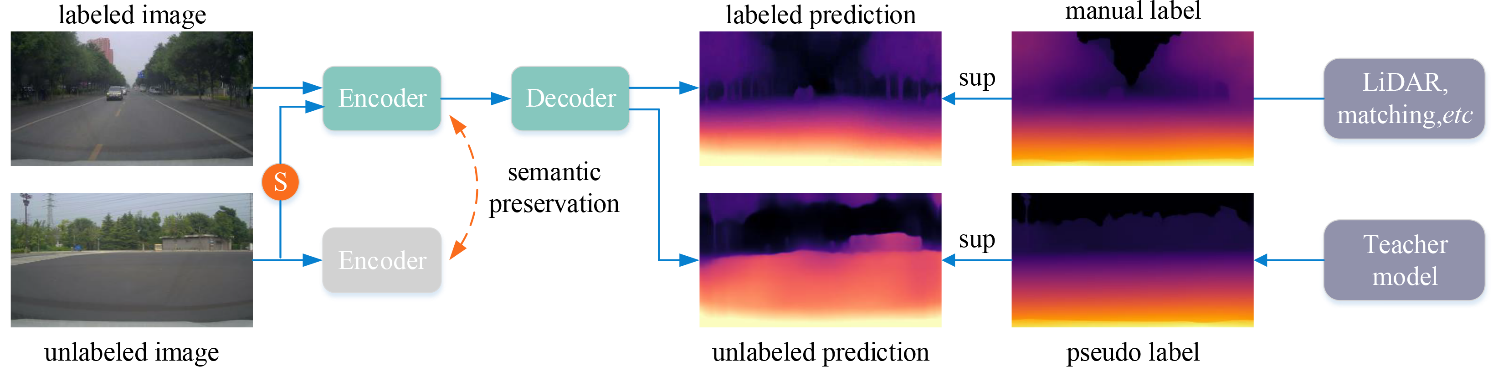


**Supplementary Figure 3.** **The training process of Depth Anything.** In the figure, $S$ represents the perturbation added to the module. The Encoder represents the network structure that extracts the original dataset information into a vector, and the module uses the VIT-Large network as the encoder. The Decoder represents the network structure that generates a depth map from the vector, and module uses the DPT decoder network as the decoder.


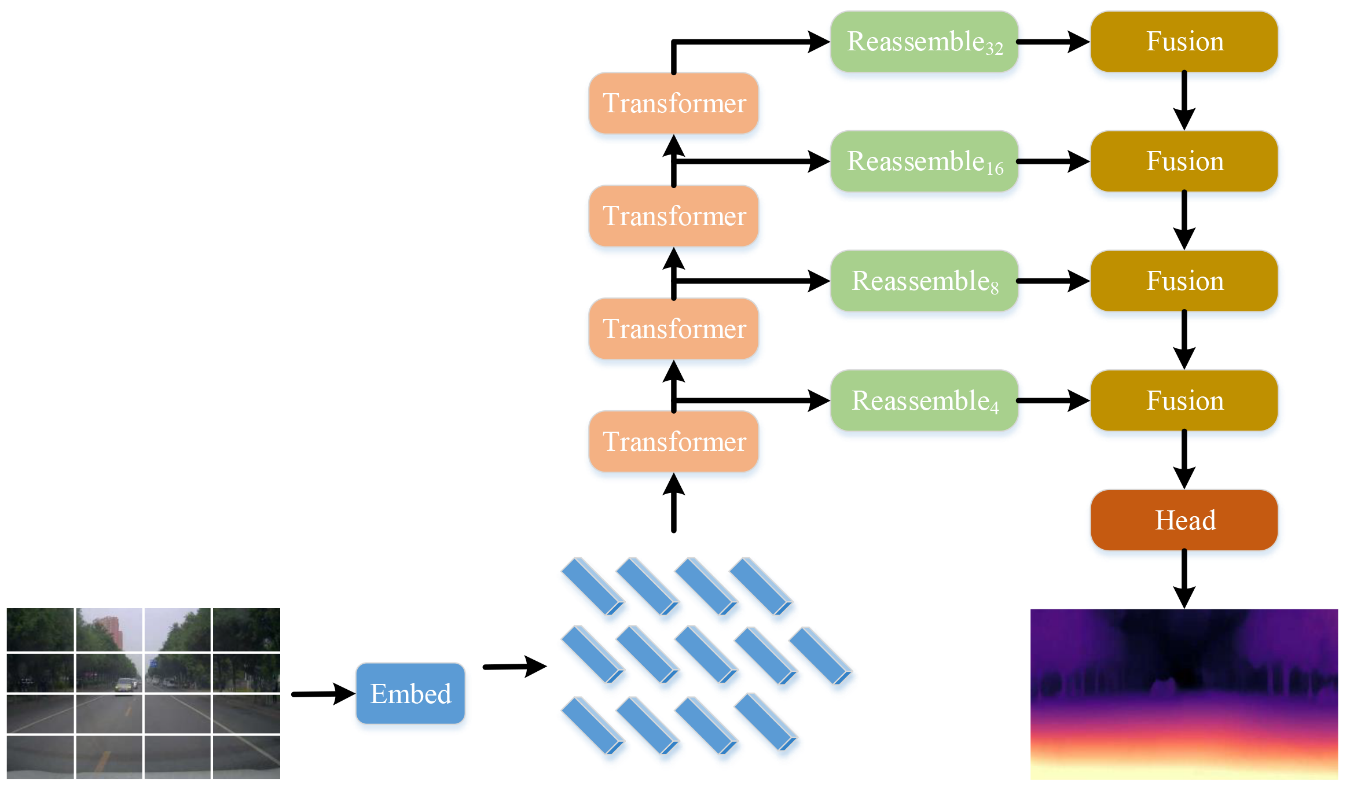


**Supplementary Figure 4. The basic architecture of the teacher or student model. This model incorporates the DINO-V2 encoder with a ViT-L backbone and the DPT decoder.**


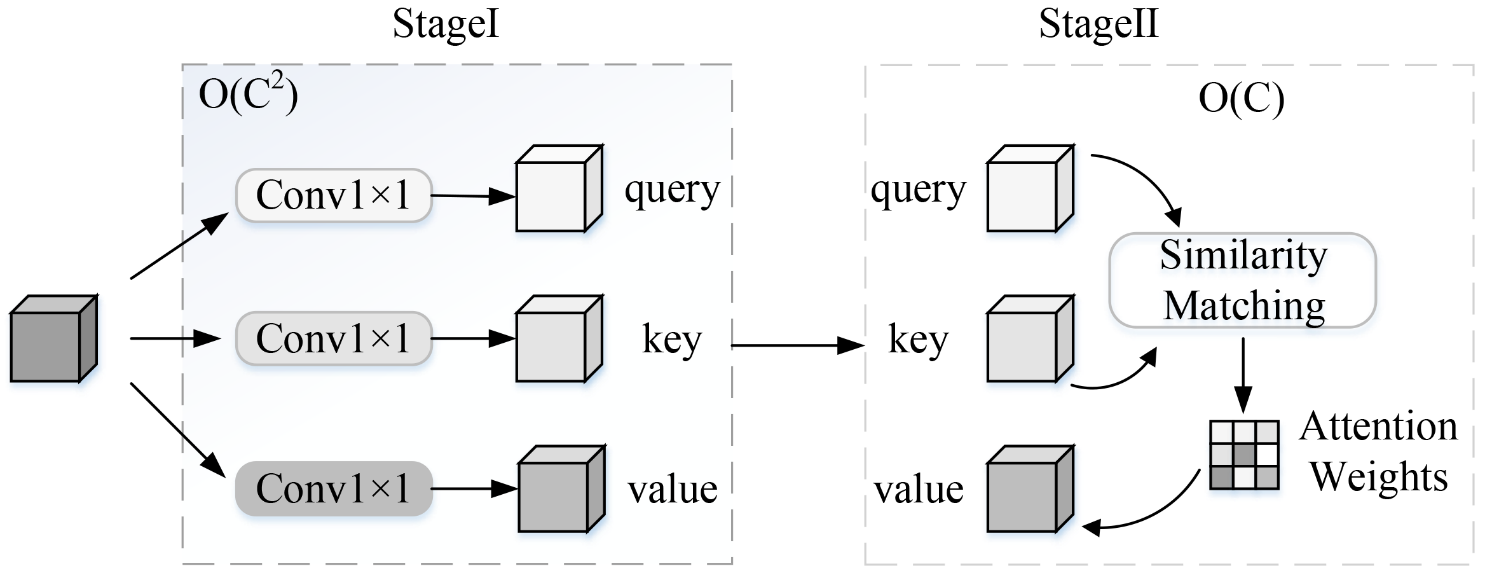


**Supplementary Figure 5.** **Self-attention module structure.** The self-attention mechanism maps the input dataset into query, key, and value matrices using 1$\times$1 convolutions. Then, it aggregates the output query and key matrices to obtain attention weights. Finally, it combines the attention weights with the value matrix.


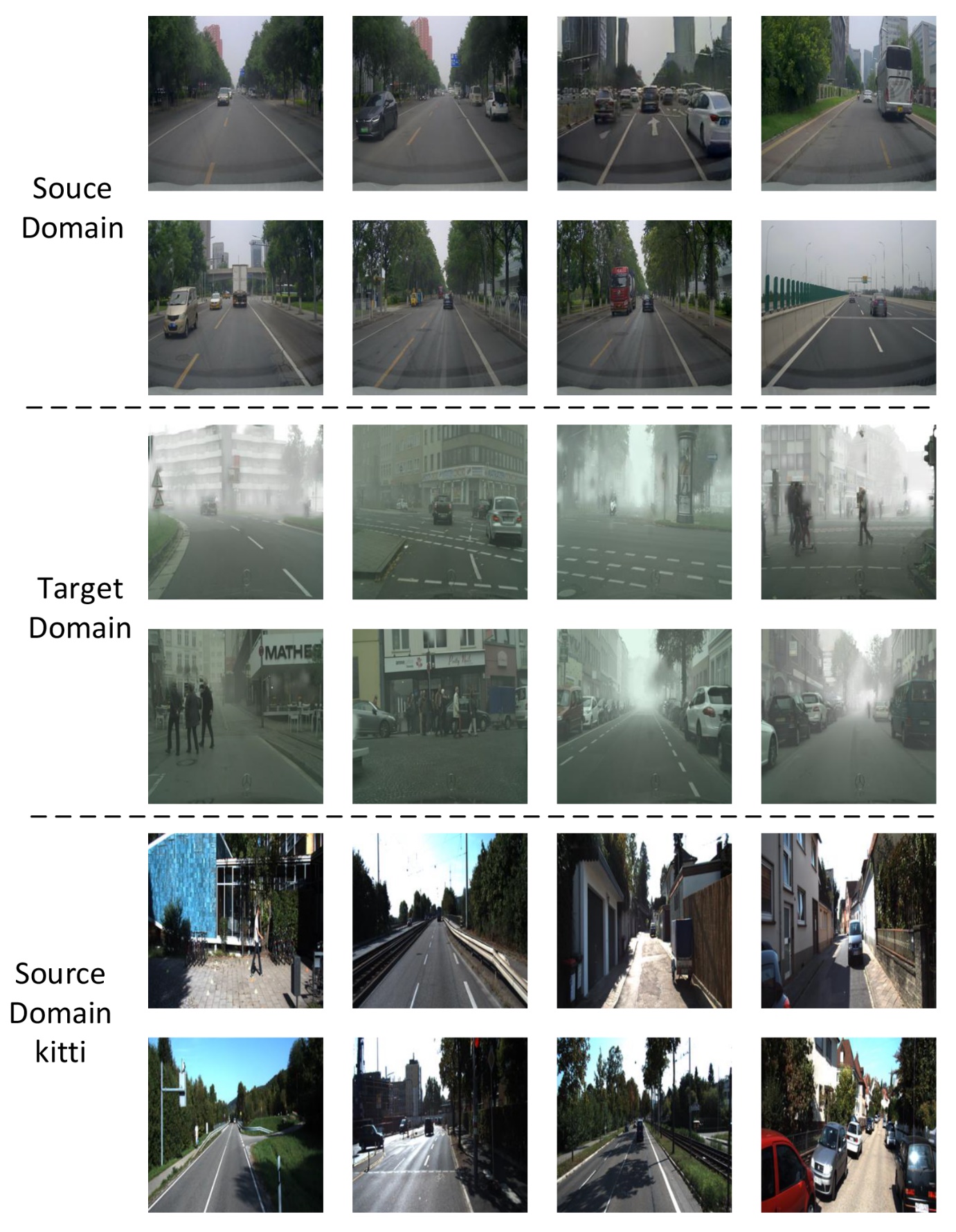


**Supplementary Figure 6. Sample displays of the source domain and target domain datasets.**


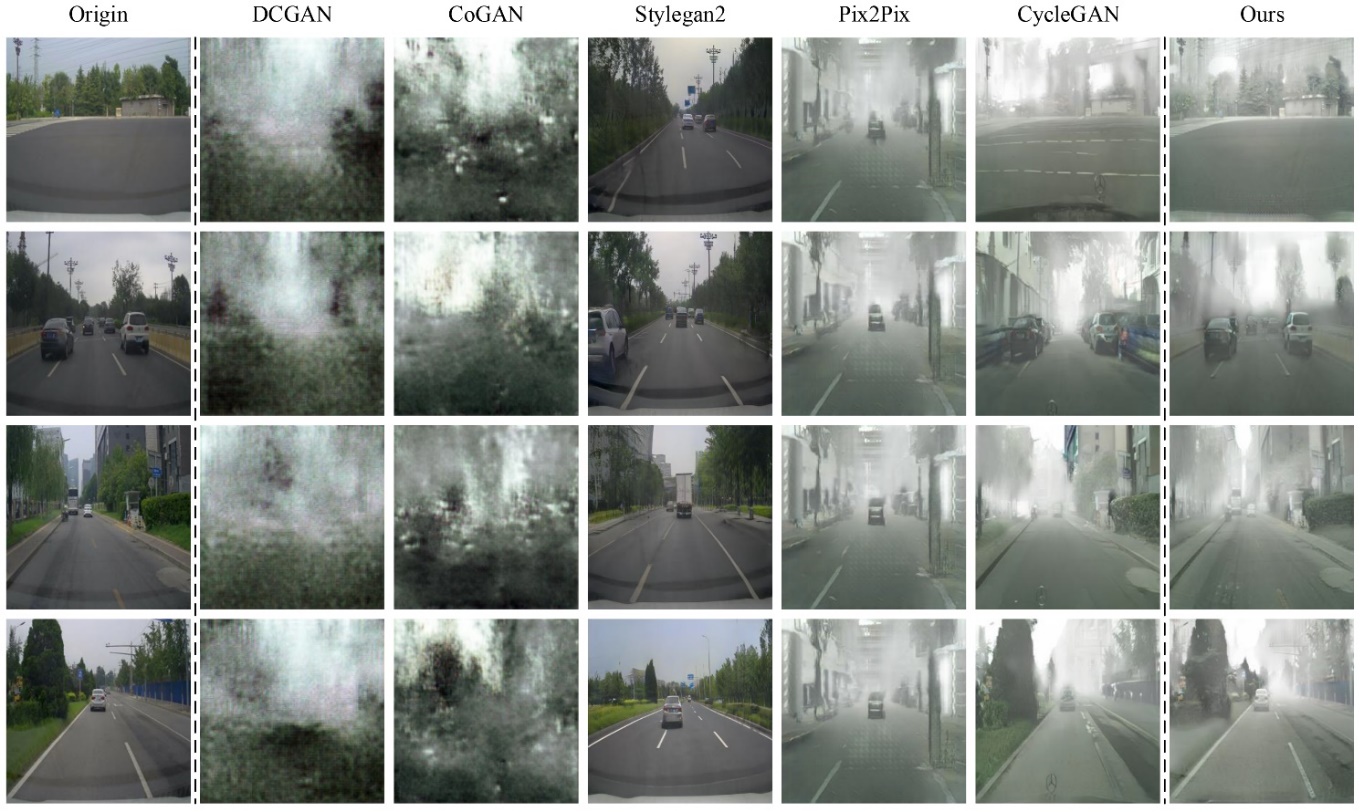


**Supplementary Figure 7.** **Multi-model effect comparison.** The images in the leftmost column come from the original dataset $X$. The images in the middle columns are generated by different GANs that have learned the style of the target domain $Y$. The images in the rightmost column are the results of our method for style transfer


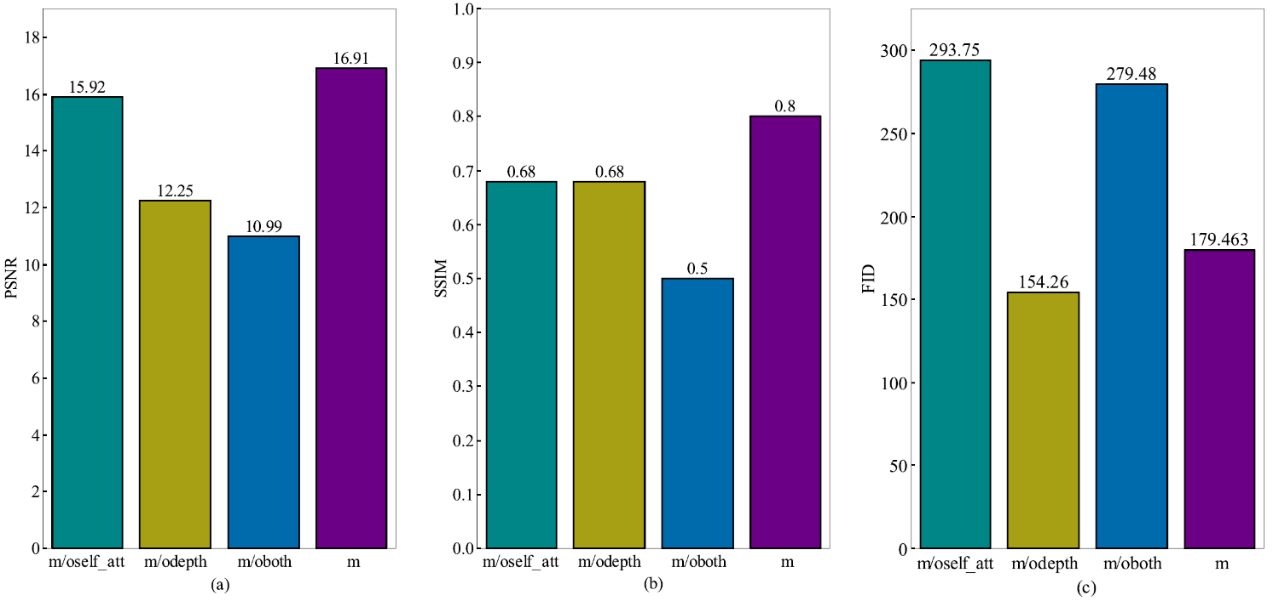


**Supplementary Figure 8.** **Ablation Experiment.** In the bar charts for PSNR and SSIM, higher values indicate better performance of the model. Conversely, in the bar chart for FID, lower values indicate better performance.


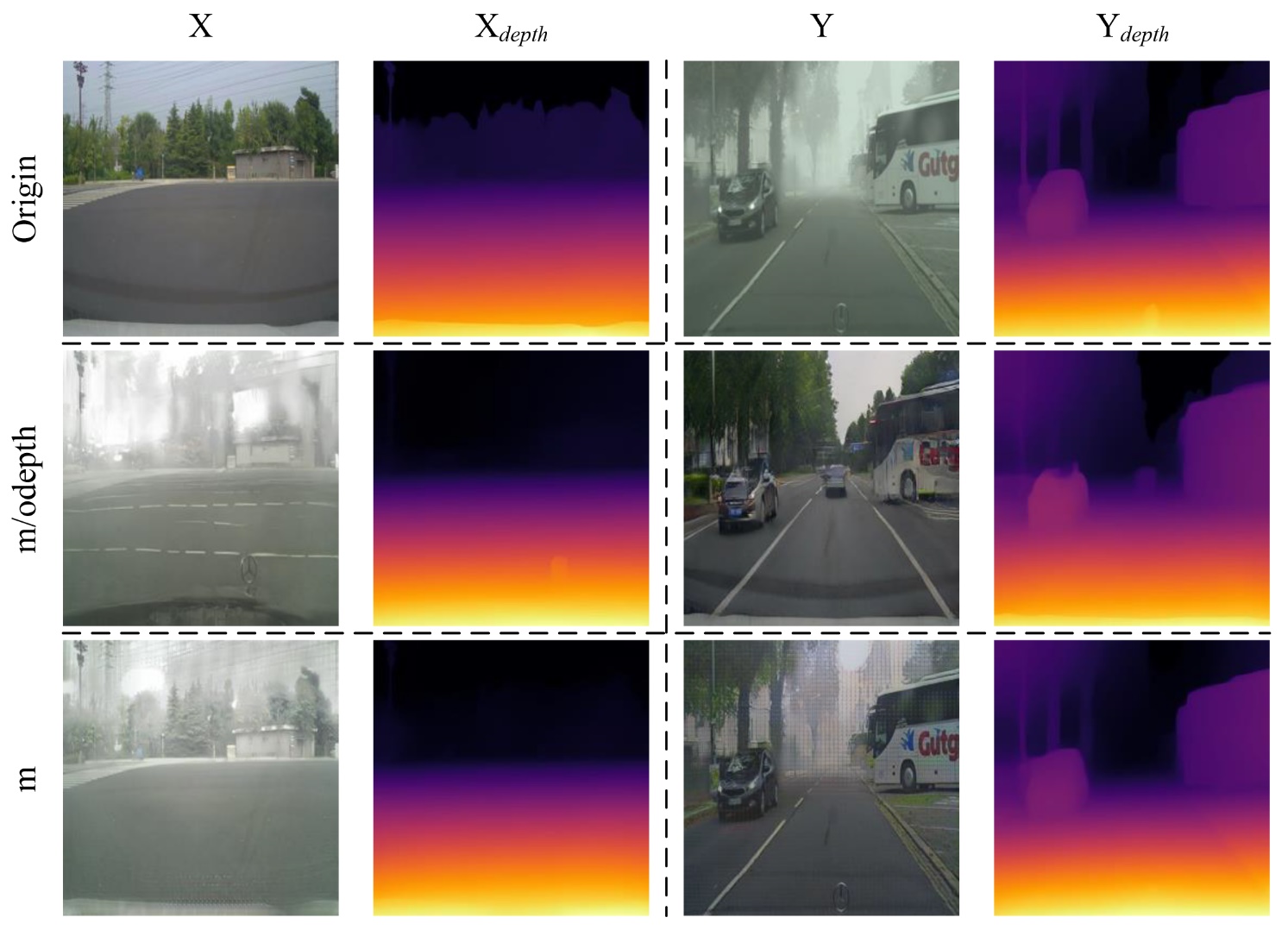


**Supplementary Figure 9.** **Comparison of ablation experiment generated graphs.** The first row of the graph displays the image from the original dataset $X$ along with the corresponding depth map, as well as the image from the original dataset $Y$ and the corresponding depth map. The second row presents the image and depth map generated after applying style transfer to image from dataset $X$ and dataset $Y$. This transformation incorporates the self-attention mechanism into the method. In the last row, the proposed method is applied to the image and depth map obtained after the style transfer of the image from dataset $X$ and dataset $Y$.


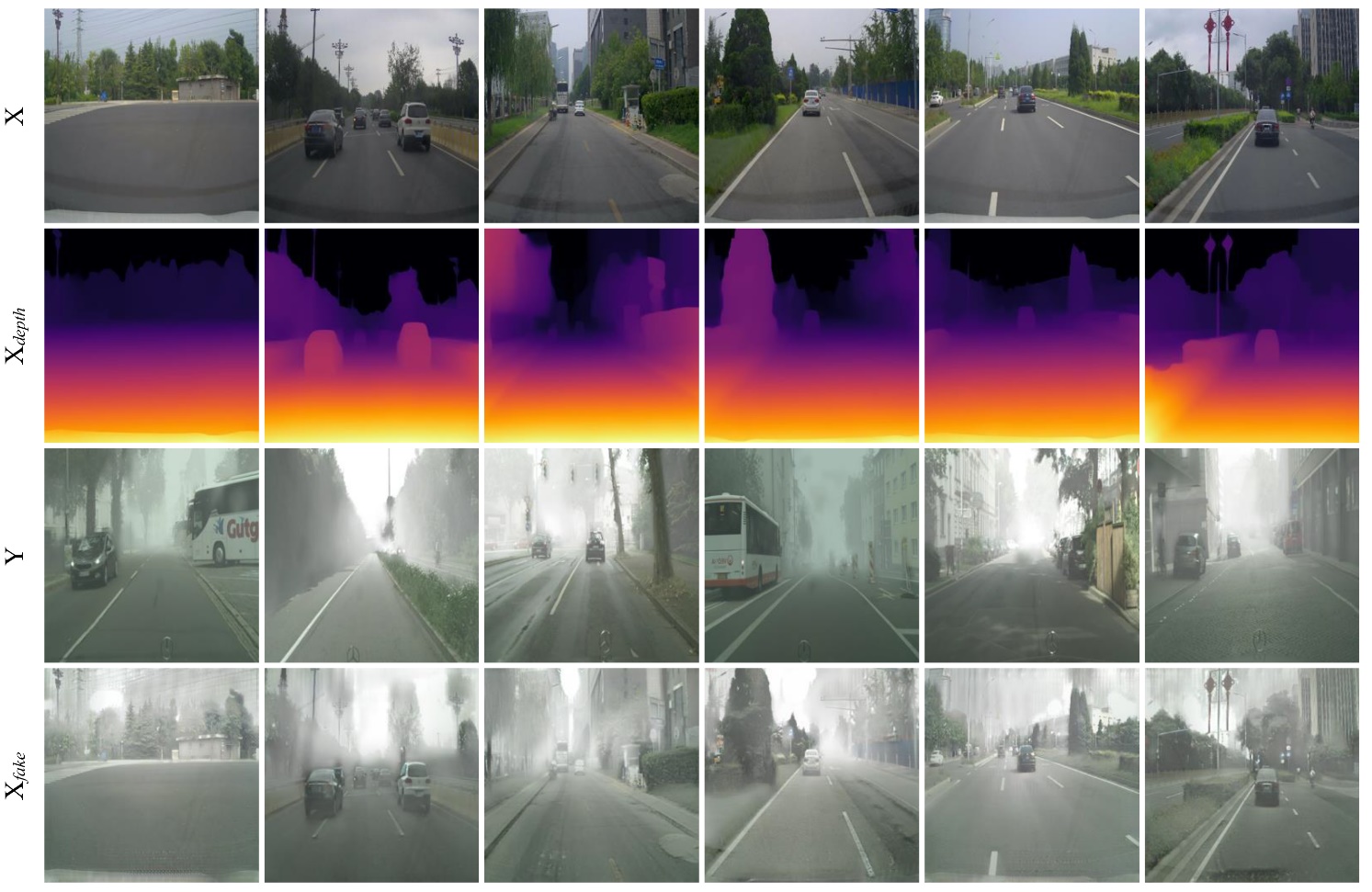


**Supplementary Figure 10.** **Presentation of pictures generated by the proposed method.** The first row displays images of different road scenes from dataset $X$. The second row shows the corresponding depth maps of the images in the first row. The third row contains images from the target domain $Y$. The last row presents foggy street scene images generated by applying the proposed method to the images from dataset $X$. This transformation is achieved by learning the style of dataset $Y$ and transferring it to the road scenes from dataset $X$.


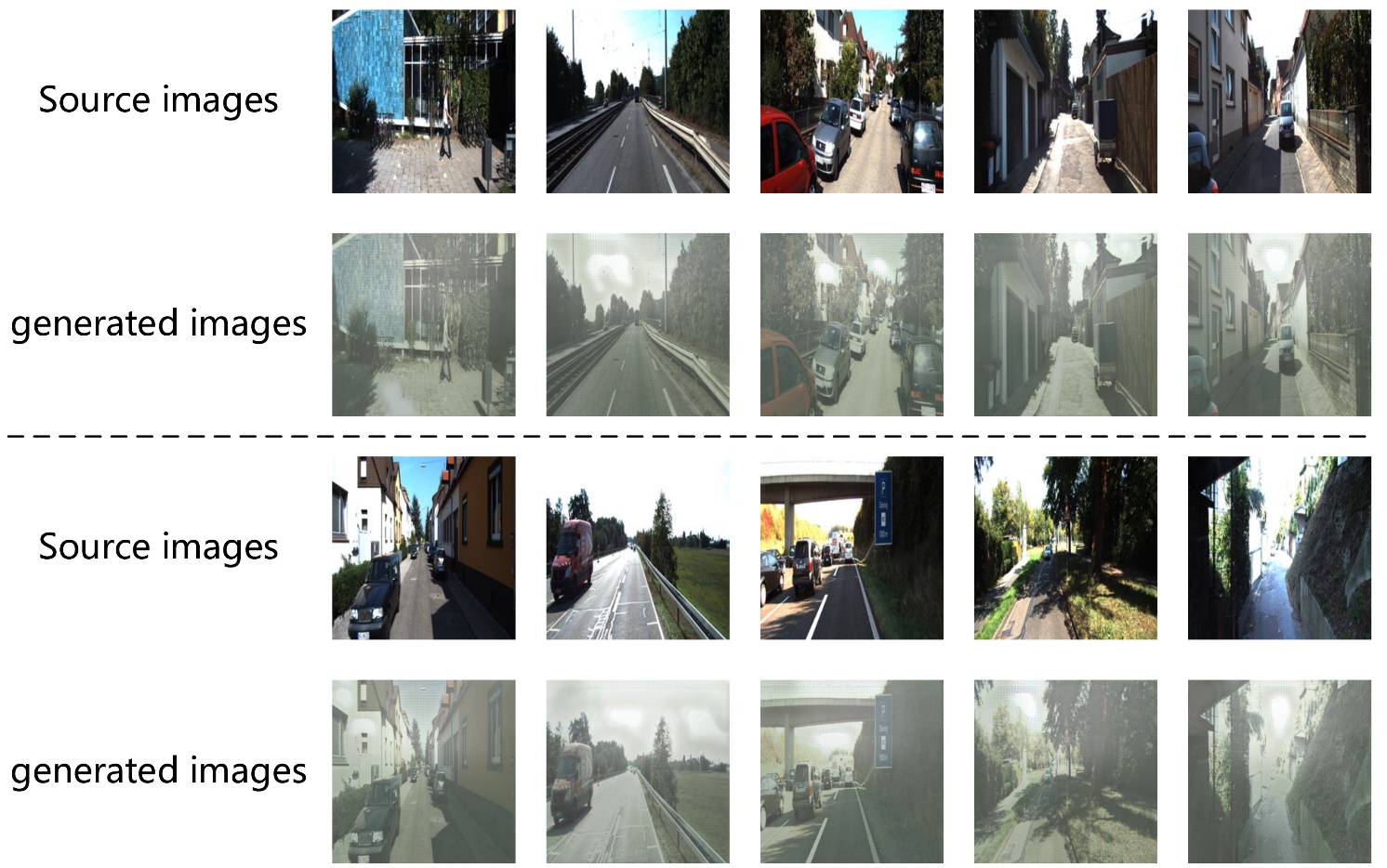


**Supplementary Figure 11 Image results generated using the KITTI dataset.**

## Supplementary Tables

**Table 1** Detailed structure of the generator branch

| **Layer** | **Elements** | **K** | **In/Out Size** | **In/Out Channels** |
| --- | --- | --- | --- | --- |
| Feature extraction Layers | Pad | - | 256/262 | 3/3 |
|  | Conv | 7 | 262/256 | 3/64 |
|  |  | 3 | 256/128 | 64/128 |
|  |  | 3 | 128/64 | 128/256 |
| Resnet Block Layers | Resnet blocks  （9×） | 3 | 64/64 | 256/256 |
| Self Attention | Self Attention | - | 64/64 | 256/256 |
| Upsample Layers | ConvTranspose | 3 | 64/128 | 256/128 |
|  | ConvTranspose | 3 | 128/256 | 128/64 |
|  | Pad | - | 256/262 | 64/64 |
|  | Conv | 7 | 262/256 | 64/3 |
|  | Tanh | - | 256/256 | 3/3 |

**Table 2** Detailed structure of the discriminator

| **Elements** | **K** | **In/Out Size** | **In/Out Channels** |
| --- | --- | --- | --- |
| Conv1 | 4 | 256/128 | 3/64 |
| Conv2 | 4 | 128/64 | 64/128 |
| Conv3 | 4 | 64/32 | 128/256 |
| Conv4 | 4 | 32/31 | 256/512 |
| Conv5 | 4 | 31/30 | 512/1 |

**Table 3.** Comparison of evalution metric results for different methods

| **Model** | **PSNR↑** | **SSIM↑** | **FID↓** |
| --- | --- | --- | --- |
| DCGAN | 13.83 | 0.31 | 462.44 |
| CoGAN | 11.95 | 0.32 | 388.06 |
| Stylegan2 | 14.34 | 0.48 | 216.80 |
| Pix2pix | 16.32 | 0.39 | 307.10 |
| Cyclegan | 10.99 | 0.50 | 279.48 |
| Ours | **16.91** | **0.80** | **179.46** |

**Table 4.** Different hyperparameter results comparison

| **Hyperparameter** | **Hyperparameter Details** | **PSNR↑** | **SSIM↑** | **FID↓** |
| --- | --- | --- | --- | --- |
| Epoch | n_epochs=50, n_epochs_decay=50 | **16.91** | **0.80** | **179.46** |
|  | n_epochs=100, n_epochs_decay=0 | 9.07 | 0.43 | 257.52 |
|  | n_epochs=0, n_epochs_decay=100 | 11.81 | 0.55 | 213.32 |
|  | n_epochs=70, n_epochs_decay=30 | 12.65 | 0.48 | 343.81 |
|  | n_epochs=30, n_epochs_decay=70 | 9.22 | 0.51 | 229.07 |
| Lambda | lambdA=1, lambdB=1 | 15.92 | 0.68 | 293.75 |
|  | lambdA=10, lambdB=10 | 12.34 | 0.64 | 290.07 |
|  | lambdA=50, lambdB=50 | 15.80 | 0.78 | 202.91 |
|  | lambdA=100, lambdB=100 | **16.91** | **0.80** | **179.46** |
| Identity | identity=0.5 | **16.91** | **0.80** | **179.46** |
|  | identity=0.25 | 15.82 | 0.74 | 188.32 |
|  | identity=0.75 | 14.37 | 0.69 | 204.72 |
